# From policy to practice: A South African clinical toolkit for SRY gene screening in sport

**DOI:** 10.17159/2078-516X/2026/v38i1a27032

**Published:** 2026-09-15

**Authors:** P Zondi, E Kotzé, JA Dave, L Gordon

**Affiliations:** 1 School of Clinical Medicine, College of Health Sciences, University of KwaZulu-Natal, Durban, South Africa; 2 Health Care Centre, North-West University, Potchefstroom, South Africa; 3 Division of Endocrinology, Department of Medicine, Faculty of Health Sciences, University of Cape Town, Cape Town, South Africa; 4 Division of Physiological Sciences and Health through Physical Activity, Lifestyle and Sport Research Centre, Department of Human Biology, Faculty of Health Sciences, University of Cape Town, Cape Town, South Africa

**Keywords:** genetic screening, sex eligibility, elite sport, athlete screening

## Abstract

The recent implementation of SRY (sex-determining region Y) gene screening by international sporting federations has introduced new clinical responsibilities for practitioners involved in athlete care. Recent policy developments, including updated guidance from the International Olympic Committee, further signal the expanding role of genetic screening in elite sport. While federation-level policies exist, there is limited clinician-focused guidance on how to operationalise screening, manage results, and ethically support athletes, particularly in low- and middle-income settings. This paper presents a practical toolkit and a step-by-step clinical pathway to support clinicians involved in SRY gene screening. Informed by clinical implementation and refined through multidisciplinary peer feedback, the proposed framework emphasises role clarity, informed consent, confidentiality, appropriate referral, and athlete-centred communication. It is intended as a clinical support tool rather than a formal guideline, consensus statement, or legal interpretation. By translating policy into a structured clinical process, the framework supports consistent, ethical, and contextually relevant practice.

In 2025, World Boxing and World Athletics introduced SRY (sex-determining region Y) gene screening as part of eligibility regulations for female competition categories, incorporating genetic screening into existing sex-based eligibility frameworks.^[1, 2]^ The SRY gene is located on the Y chromosome and plays a key role in male sex development.^[3]^ At present, SRY screening applies primarily to elite athletes competing in designated international events. In March 2026, the International Olympic Committee announced an updated policy confirming SRY gene screening as the basis for determining eligibility in the female category at the Olympic Games, effective from Los Angeles 2028.^[4]^ Similar approaches may be considered by other International Federations.

Although policy documents and athlete-facing explanatory resources are available in federations where SRY screening has been implemented, clinicians are often asked to translate these requirements into clinical practice in the absence of structured, country-specific guidance. General practitioners, sports and exercise medicine physicians, and team doctors may be approached to explain screening requirements, obtain consent, coordinate laboratory testing, or support athletes after results are received. The lack of locally contextualised clinical guidance is particularly relevant in low- and middle-income countries (LMICs), where access to specialist genetic services is variable and clinicians are often the first and only point of contact.

Against this background, this paper presents a practical toolkit and a step-by-step clinical pathway to support South African healthcare professionals in implementing SRY gene screening. This framework is intended as a clinical support tool rather than a formal guideline, consensus statement, or legal interpretation.

## Rationale for a physician-led framework

Although SRY gene screening is used within eligibility frameworks in elite sport, it does not constitute a diagnostic evaluation for differences of sex development (DSD), nor does it independently determine athletic performance potential.^[3]^ Rather, it functions as a screening tool; therefore, a positive test result may raise complex clinical and psychosocial considerations, including the need for further investigation, specialist referral, and sensitive communication.^[5]^

In practice, team doctors occupy a critical intermediary role between athletes, specialists, laboratories and sports federations. Without clear role definition and procedural guidance, there is a risk of inconsistent practice, inappropriate disclosure, and avoidable athlete distress. Therefore, a structured clinical framework is needed to guide physicians in managing these responsibilities with clarity, ethical integrity, and due regard for athlete welfare.

## Development of the clinical toolkit and pathway

The framework was developed following early clinical experience with implementing SRY gene screening ahead of the 2025 World Athletics Championships. Initial versions were presented at the South African College of Sport and Exercise Medicine (CSEM) Conference in 2025 and were subsequently refined through multidisciplinary peer feedback and follow-up discussions. Further structured feedback was obtained from independent clinicians across sports and exercise medicine, endocrinology, genetics, gynaecology, and athlete care contexts, with revisions focused on ethical implementation, role clarity, confidentiality, referral pathways, and contextual feasibility.

The emphasis throughout is on preparedness, ethical conduct, and athlete-centred communication within a complex and evolving policy environment, rather than eligibility adjudication, which remains the responsibility of sporting federations.

## Clinical toolkit: Preparing for SRY gene screening

Table 1 presents a clinician readiness toolkit that outlines the key domains clinicians should address before engaging in SRY screening. This toolkit promotes clinician preparedness rather than reactive engagement, recognising that many clinicians may encounter SRY screening for the first time through athlete enquiry.

## Step-by-Step clinical pathway

To complement the readiness toolkit, a structured clinical pathway has been developed to guide physicians from initial athlete presentation through to post-result support. This pathway is presented below:

### 1. Initial consultation

- The athlete presents for a pre-participation, competition-related, or advisory consultation. The physician explains the purpose of SRY screening, its once-off nature, and the federation’s role in eligibility determination, including possible outcomes and implications for eligibility and clinical follow-up.
- Athletes should be counselled that SRY screening is not a diagnostic test and that further assessment may be required to clarify the clinical significance of a positive result.
- Discussion should also acknowledge the potential psychosocial, cultural, and emotional considerations that may arise during the wait for a result or referral, particularly after a positive screening.
- General educational information may be delivered in a group setting; however, informed consent and individual discussion should be conducted on a one-on-one basis.
- Athletes should be provided with written information explaining the purpose of screening, possible outcomes, implications for eligibility, confidentiality arrangements, and available support services prior to consenting to testing.
- Consultations are ideally delivered in person, but if unavoidable, can be delivered via secure remote platforms, subject to confidentiality safeguards.

### 2. Informed consent

- Written informed consent is obtained following discussion of the purpose of screening, potential implications for eligibility, limitations of the test, confidentiality arrangements, disclosure pathways, and options for further support where required.
- Obtain consent to share results with the federation medical manager.

### 3. Clinical assessment

- The extent of clinical assessment should be context-specific.
- Where SRY screening is conducted as part of a broader pre-participation health evaluation, relevant history and examination findings may already be available to the treating physician.
- Where screening is undertaken solely to comply with eligibility regulations, additional physical examination is not routinely required. Further evaluation should be reserved for positive results or for specific clinical concerns and undertaken by an appropriately trained specialist (e.g., an endocrinologist or a gynaecologist).

### 4. Laboratory referral

- Prior to initiating screening, clinicians should confirm access to an accredited PCR-capable laboratory and any federation-specific testing requirements.
- Referral is made to an accredited PCR-based laboratory.
- Where screening is required for competition eligibility, confirm any federation-specified sample-handling and identity-verification requirements with the laboratory.
- Where local laboratory access is limited, clinicians should consider referral to the nearest accredited PCR-capable laboratory or consult the relevant federation regarding approved testing pathways, which may include testing at an alternative location or competition venue.

### 5. Results management

- Results are communicated confidentially to the athlete by the treating physician.
- SRY-negative results confirm eligibility under current federation rules.
- SRY-positive results should be interpreted within the broader clinical context with prompt follow-up consultation and referral to an endocrinologist or gynaecologist.

### 6. Reporting and record keeping

- Clinical records, consent documentation, and laboratory reports should be stored in secure, access-controlled systems in accordance with national health record retention and data protection legislation (e.g., POPIA). Electronic records should be password-protected and maintained on secure servers compliant with institutional data protection standards.
- Access to genetic results should be restricted to authorised medical personnel. Disclosure to federations should occur with the athlete’s explicit informed consent and in accordance with applicable federation policy, sharing only the minimum necessary information for eligibility purposes. Disclosure to any third parties should occur only at the athlete’s direction and with documented informed consent. Clinical genetic reports remain part of the confidential medical record.

### 7. Athlete support

- Ongoing support should be offered where appropriate, including referral for genetic counselling and psychological support. Where further clinical evaluation is indicated, referral to an appropriately trained specialist (e.g., endocrinologist or gynaecologist) should be arranged.
- Where local specialist services are unavailable, support may be facilitated through regional tertiary centres, remote specialist consultation, or federation-supported referral, where available.
- When screening is conducted under federation regulations, access to professional support services may be facilitated through federation medical channels. Responsibility for the costs of further assessment should be clarified in advance and may vary depending on federation policy, healthcare setting, and funding arrangements.

This pathway provides a structured approach for clinicians to manage SRY gene screening within their professional responsibilities, recognising that eligibility decisions fall within federation governance frameworks, under which screening is conducted in accordance with formal competition regulations. Where screening is initiated outside federation processes (e.g., at the request of a professional athlete or representative), elements related to reporting, federation disclosure, and governance-linked procedures may not apply; however, the principles of informed consent, confidentiality, appropriate referral, and athlete-centred care remain unchanged.

## Implications for clinical practice

The introduction of SRY gene screening into elite sport requires physicians to navigate a new intersection between regulatory compliance and clinical care. This includes eligibility-linked screening, obtaining informed consent, managing sensitive results, and coordinating appropriate referral pathways while maintaining ethical, athlete-centred care. This complexity is further shaped by recent evolving eligibility regulations.

Clinicians involved in SRY screening may experience tension between their role as an athlete advocate and their role in facilitating compliance with federation regulations. While eligibility decisions remain the responsibility of sporting federations, clinicians should prioritise transparent communication, informed decision-making, confidentiality, and athlete welfare throughout the process. Where concerns arise about conflicts of interest or a clinician’s ability to provide independent advice, referral to another appropriately qualified practitioner may be considered.

The toolkit and pathway presented in this paper provide a structured framework to help physicians consistently and ethically meet these responsibilities. By emphasising preparedness, role clarity, informed consent, confidentiality, and athlete-centred communication, the framework provides an operationally feasible approach within the South African healthcare context and may be adapted to other settings, subject to local regulatory, clinical, and resource considerations.

## Conclusion

This paper provides a structured clinical framework to support physicians navigating SRY gene screening in elite sport. As eligibility regulations, scientific understanding, and international policy continue to evolve, this toolkit should be regarded as a living document, informed by implementation experience and ongoing developments in sport governance.

## Supplementary Information



## Figures and Tables

**Table 1:** Clinician readiness toolkit for SRY gene screening

Domain	Key considersations
**Knowledge**	Understanding federation-specific SRY screening policies; basic principles of SRY screening and interpretation; awareness of clinical indicators warranting specialist referral
**Accredited laboratory**	Identification of a local PCR-capable laboratory; confirmation of reporting standards, turnaround times and pre-analytical requirements (including sample type and collection logistics)
**Clinical network**	Pre-identified multidisciplinary referral network for activation in the event of a positive SRY screening result. Network should include endocrinologist, gynaecologist, geneticist, psychologist or social worker, and federation medical contacts
**Documentation and consent**	Availability of SRY-specific informed consent; POPIA-compliant data storage; separation of clinical records from federation administrative documentation
**Communication readiness**	Clear, non-stigmatising explanations for athletes; emphasis on the once-off nature of screening, confidentiality, fairness, and athlete welfare

SRY, sex-determining region Y
