# Supplementary material for: From policy to practice: A South African clinical toolkit for SRY gene screening in sport
Source: S Afr J Sports Med. 2026 Sep 15;38(1):v38i1a27032. doi: 10.17159/2078-516X/2026/v38i1a27032 (PMC13644805; doi:10.17159/2078-516X/2026/v38i1a27032)
Supplement: Supplementary file 1 [file 2078-516X-38-v38i1a27032-s001.pdf]

From policy to practice: A South African clinical toolkit for SRY gene screening in sport

Supplementary material 1: Athlete Information Sheet – SRY Gene Screening

This information sheet is intended to help you understand:
- why screening may be requested,
- what the process involves,
- possible outcomes of screening,
- and how your information and results will be managed.

What is SRY gene screening?

SRY gene screening is a genetic test used to determine whether the SRY (Sex-determining Region Y) gene is present. The SRY gene is found on the Y chromosome and plays an important role in male sex development.

The test is performed on a blood sample, saliva sample or cheek swab. In most cases, SRY screening only needs to be performed once.

Why am I being asked to do this screening?

Some sporting federations now require SRY gene screening for athletes who wish to compete in the female category at certain elite international competitions.

Requirements differ between sports and federations and may apply to:
- World Ranking competitions,
- international championship events,
- or competitions where performances may count towards national rankings or records.

Eligibility rules are determined by the relevant international sporting federation, not by your coach, local club, doctor or healthcare team.

Who currently requires screening?

At present, SRY screening applies mainly to elite-level athletes competing in specific international competitions under the regulations of certain sporting federations.

Not all athletes, competitions, or sports currently require screening. Your federation or team medical staff should explain whether these requirements apply to you.

What does the screening involve?

- This is a once-off screening test under most federation regulations.
- Screening is usually performed using:
  - a cheek swab,
  - saliva sample, or
  - a blood sample.
- The test is processed in an accredited laboratory.

What are the possible outcomes?

<u>SRY-negative result</u>

This means that no SRY gene was detected. Under current federation rules, this will confirm eligibility for participation in the female competition category. In most cases, no further SRY screening will be required unless there is reason to question the accuracy of the original result.

<u>SRY-positive result</u>

This means that the SRY gene was detected.

An SRY-positive result:
- does not on its own establish a medical diagnosis,
- does not automatically indicate a health problem,
- may lead to recommendations for further medical assessment or specialist referral.

Under current federation regulations, an SRY-positive result may affect eligibility for participation in the female competition category pending further review.
Further assessment, where needed, may involve referral to specialists such as an endocrinologist, gynaecologist, clinical geneticist, counselling psychologist or social worker.

Possible emotional and psychological responses
Athletes may experience a range of emotional responses during or after the screening process. These can include anxiety, uncertainty, concern about competition eligibility, confusion about the meaning of results, or worry about who may have access to their information.

These reactions are understandable, particularly while waiting for results or if further medical assessment is recommended. Athletes are encouraged to discuss any concerns with their treating doctor, team medical staff, or other appropriate support professionals.

What happens to my information/results?
Your screening results are treated as confidential medical information.

Clinical records and laboratory results will be stored securely and managed in accordance with applicable privacy and data protection legislation, as well as relevant federation policies.

Results will only be shared with authorised individuals involved in your medical care or eligibility process, and disclosure should occur in accordance with your informed consent and applicable federation requirements.

You should feel comfortable asking:

- who will have access to your results,
- how your information will be stored,
- and what information may need to be shared with your sporting federation.

Who can I speak to if I have questions/support needs?
You are encouraged to ask questions at any stage of the process.

Support may be available through:

- your treating doctor,
- team doctor or medical staff,
- an endocrinologist or gynaecologist,
- a clinical geneticist or genetic counsellor,
- a psychologist or counsellor,
- or federation medical support services where available.

If further assessment or follow-up is needed, your doctor should discuss this with you, confirm who is responsible for the costs of care, and help guide appropriate referral and support pathways.

Do I have to do this screening?
No. SRY gene screening is voluntary and requires your informed consent.

Before deciding, you should receive information about:

- why screening is being requested,
- what the process involves,
- possible outcomes,
- and how your results will be managed.

You may choose not to undergo screening or withdraw your consent at any time.

However, athletes should understand that under current federation regulations, declining or withdrawing consent for SRY screening may affect eligibility to compete in the female category at certain competitions or have performances recognised for ranking or record purposes.

What is the role of my doctor?
Your doctor’s role is to:

- explain the screening process,
- answer questions,
- obtain informed consent,

- coordinate screening where appropriate,
- communicate results confidentially,
- and arrange further medical assessment or support if needed.

Your doctor does not decide competition eligibility. Eligibility decisions are made by the relevant sporting federation according to its regulations.

If you have questions or concerns at any stage, you are encouraged to discuss them with your doctor or healthcare team.

Supplementary material 2: Clinician Information Sheet – SRY Gene Screening

Purpose and scope

SRY gene screening is used by some international sporting federations to help determine eligibility for participation in the female category at certain elite competitions. The presence or absence of the SRY (Sex-determining Region Y) gene functions as a screening marker within eligibility frameworks rather than as a diagnostic medical investigation.

Eligibility requirements and implementation processes may vary between sports, federations, and competition levels.

Key clinical principles

- SRY gene screening does not diagnose differences of sex development (DSD)
- SRY gene screening does not assess athletic performance
- An SRY-positive result does not independently determine eligibility
- Interpretation should occur within the relevant federation policy and clinical context
- Eligibility determinations remain the responsibility of the relevant sporting federation

SRY screening and Y chromosome material

The SRY (Sex-determining Region Y) gene is usually located on the Y chromosome and plays a key role in male sex development. Within current sporting eligibility frameworks, SRY screening is used as a marker for the presence of Y chromosome material and functions as an initial screening step rather than a standalone determinant of eligibility.

Clinical relevance beyond eligibility

While SRY gene screening is used within sporting eligibility frameworks, the identification of Y chromosome material may have broader clinical relevance in selected cases. In some individuals, this may be associated with differences of sex development (DSD) and implications for reproductive health, hormonal function, bone health, or longer-term medical follow-up.

An SRY-positive result alone does not establish a diagnosis and should be interpreted within the broader clinical and sporting context, including consideration of further assessment or specialist referral where appropriate.

SRY screening undertaken for eligibility purposes does not routinely require a full physical or genital examination by the requesting clinician. Clinical assessment should be guided by the presenting context and conducted respectfully and within the clinician's scope of practice. Where further specialist evaluation is indicated, referral to an endocrinologist, gynaecologist, or other appropriately experienced specialist should be considered.

Limitations of SRY Screening

Clinicians should recognise that SRY screening is a screening tool rather than a diagnostic test. Although the presence of the SRY gene may indicate Y chromosome material, SRY status alone does not determine phenotype, hormonal profile, athletic performance, or eligibility.

False-positive and false-negative results may occur in rare circumstances, including mosaicism, translocation events, or differences of sex development. Consequently, SRY results should not be interpreted in isolation. Positive results require appropriate clinical review and specialist referral where indicated, while eligibility determinations remain the responsibility of the relevant sporting federation.

Clinician responsibilities

Clinicians involved in SRY screening may be asked to:

- Explain the purpose, limitations, and possible implications of screening
- Discuss potential emotional and psychological responses to screening and facilitate appropriate support where required
- Obtain informed consent, including discussion of confidentiality, data handling, and disclosure pathways
- Refer athletes to an accredited PCR-capable laboratory for sample collection and screening
- Provide post-result consultation and facilitate specialist referral where indicated
- Support athletes through the screening process using respectful, athlete-centred communication

Where local specialist services are unavailable, clinicians should seek support through regional tertiary centres, remote specialist consultation, or federation medical structures where available. In some cases, federations may facilitate referral to an appropriate centre for further assessment and management. The composition of the multidisciplinary team may vary according to local resource availability.

Eligibility determinations remain the responsibility of the relevant sporting federation and not the treating clinician.

Reporting, confidentiality, and support

SRY screening results constitute confidential medical information. Reporting and disclosure should occur in accordance with athlete consent, applicable privacy legislation, and relevant federation policies.

Results may be communicated through the laboratory, treating physician, or federation medical structures, depending on the relevant process and athlete consent. Access to genetic information should be restricted to individuals with a defined medical role or others specifically authorised by the athlete.

Ongoing support should be available, including counselling and specialist referral where appropriate. Where screening is conducted under federation regulations, access to additional professional support services may be facilitated through federation medical channels.

Key references and resources

1. International Olympic Committee. IOC Policy on the Protection of the Female (Women's) Category in Olympic Sport and Guiding Considerations for International Federations and Sports Governing Bodies. 2026.
2. World Athletics. World Athletics introduces SRY gene screening for athletes wishing to compete in the female category. 2025.
3. Wise J. What is the SRY gene test, and should it be used to test female athletes? BMJ. 2025;390:r1698.
4. Bermon S, Coleman DL, Handelsman DJ. SRY gene screening in elite Athletics: rationale, science and implementation of the World Athletics Regulations.
5. Zondi P, et al. From Policy to Practice: A South African Clinical Toolkit for SRY Gene Screening in Sport. Submitted manuscript.

Supplementary material 3: Informed Consent for Sport-related SRY Gene Screening

Athlete name: ____

Date of birth: ____

Email Address:____

Contact number:____

Sport / Federation: ____

Clinician name: ____

Email address:____

Contact number:____

Date: ________________

Reason for SRY Gene Screening

Please indicate the context in which SRY gene screening is being requested:

☐ **Athlete-initiated screening**
Screening requested voluntarily by the athlete and/or athlete representative outside a formal federation eligibility process.

☐ **Federation-required screening**
Screening requested or required under the regulations of a sporting federation for participation in designated competitions or eligibility for the female category.

Federation (if applicable): __________________________________

Competition/Event (if applicable): ____________________________

1. Purpose of screening

The SRY gene test detects the presence of the Sex-determining Region Y (SRY) gene on the Y chromosome, acting as a rapid, one-time check for Y-chromosomal DNA.

In the context of elite sport, SRY gene screening is performed as part of eligibility regulations for participation in certain elite level and world ranking sporting competitions and functions as a screening test within these frameworks.

2. Nature and limitations of the test

I understand that:

- The test is not a diagnostic medical test
- The test does not assess athletic performance
- A positive result does not on its own establish a medical diagnosis

3. Possible outcomes and implications

I understand that:

- A negative result may confirm eligibility under current federation rules
- A positive result
  - may prompt further clinical consultation or referral, and
  - may have implications for competition eligibility
- Eligibility decisions are made by the relevant sporting federation, not by my physician/doctor

4. Sample collection

I consent to the collection of a biological sample (buccal swab or blood) for SRY gene testing by an accredited laboratory.

5. Confidentiality and disclosure

I understand that:

- My results are confidential medical information
- My information will be stored securely and handled in line with applicable privacy legislation
- Results will be received by the doctor who ordered the test
- Results will be shared with me first
- Results will not be shared without my documented consent
- Once I have provided written consent, results will be shared in accordance with federation policy

6. Voluntary consent

I confirm that the test has been explained to me, I have had the opportunity to ask questions, and I give my consent voluntarily.

7. Additional Notes by Clinician

____

____

Athlete signature: ____________________________

Date: _______________________________________

Clinician signature: __________________________

Date: _______________________________________

Witness Name:_______________________________

Witness Signature:___________________________

Date:____
